# Chrono-communication and cardiometabolic health: The intrinsic relationship and therapeutic nutritional promises

**DOI:** 10.3389/fendo.2022.975509

**Published:** 2022-09-13

**Authors:** Pamela Senesi, Anna Ferrulli, Livio Luzi, Ileana Terruzzi

**Affiliations:** ^1^ Department of Biomedical Sciences for Health, Università degli Studi di Milano, Milan, Italy; ^2^ Department of Endocrinology, Nutrition and Metabolic Diseases, IRCCS MultiMedica, Milan, Italy

**Keywords:** Circadian disruption, cardiac clock, microbiota, food intake, time restricted feeding

## Abstract

Circadian rhythm, an innate 24-h biological clock, regulates several mammalian physiological activities anticipating daily environmental variations and optimizing available energetic resources. The circadian machinery is a complex neuronal and endocrinological network primarily organized into a central clock, suprachiasmatic nucleus (SCN), and peripheral clocks. Several small molecules generate daily circadian fluctuations ensuring inter-organ communication and coordination between external stimuli, i.e., light, food, and exercise, and body metabolism. As an orchestra, this complex network can be out of tone. Circadian disruption is often associated with obesity development and, above all, with diabetes and cardiovascular disease onset. Moreover, accumulating data highlight a bidirectional relationship between circadian misalignment and cardiometabolic disease severity. Food intake abnormalities, especially timing and composition of meal, are crucial cause of circadian disruption, but evidence from preclinical and clinical studies has shown that food could represent a unique therapeutic approach to promote circadian resynchronization. In this review, we briefly summarize the structure of circadian system and discuss the role playing by different molecules [from leptin to ghrelin, incretins, fibroblast growth factor 21 (FGF-21), growth differentiation factor 15 (GDF15)] to guarantee circadian homeostasis. Based on the recent data, we discuss the innovative nutritional interventions aimed at circadian re-synchronization and, consequently, improvement of cardiometabolic health.

## Overview of chronobiology

**Figure 1:**
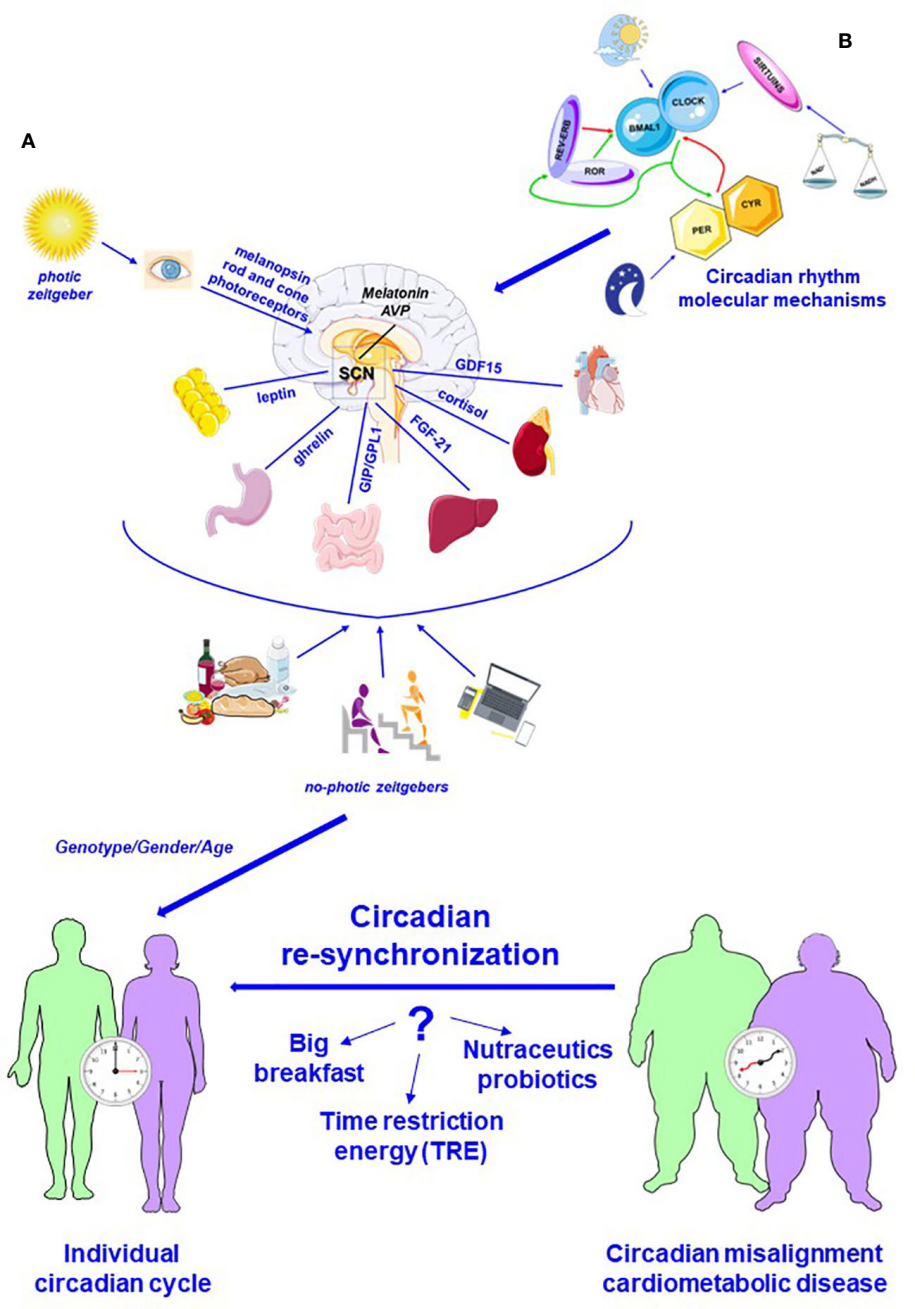
Schematic representation of the circadian system. **(A)** Circadian system is a complex communication network that allows to combine different signaling, photic and no-photic zeitgebers, maximizing the use of resources and safeguarding the survival. Light, converted into a neural input by melanopsin and rod and cone photoreceptors, is the fundamental activator of central circadian pacemaker, suprachiasmatic nucleus (SCN). Melatonin, secreted by pineal gland, is a main mediator of SCN action, which is also influenced by food, exercise, and social activities (no-photic zeitgebers). Several biomolecules, synchronizing signal, ensure the bidirectional relationship between master clock and peripheral organs. **(B)** Circadian rhythm is regulated by a complex molecular system in master and peripheral clocks. The fundamental player of this complex molecular system is represented by heterodimeric complexes CLOCK-BMAL1 that enhances daytime expression of PER and CRY factors, which translocate into the nucleus and suppress CLOCK–BMAL1 activity. During the night, PER and CRY are degraded, and a new cycle begins. Additionally, REV-ERB and ROR are also circadian regulator that respectively represses and enhances *BMAL1* expression. Additionally, sirtuins, whose activity is directly correlated to NAD^+^/NADH, regulate CLOCK-BMAL1 action. In addition, genetic components, gender, and age influence circadian rhythms and contribute to define individual circadian cycle. This complex network can break down, and circadian misalignment is an important risk factor for the development of cardiometabolic pathologies. Recent data highlight that nutrition could be a crucial metronome able to lead circadian re-synchronization: high-calorie breakfast associated with reduced food intake at dinner, supplementation with nutritional compounds, and, above all, time-restricted energy (TRE) could have beneficial action counteracting excessive weight gain and protection from cardiometabolic diseases (modified by Servier Medical Art by Servier is licensed under a Creative Commons Attribution 3.0 Unported License).

Most organisms, from bacteria to plants and humans, coordinate their physiological function and behavior with the fluctuating environment in a 24-h daily cycle (1–3). This biological daily rhythm, called circadian rhythm from Latin words *circa dies* (meaning approximately day), is the adaptative response to Earth’s rotation in order to maximize the use of existing resources ensuring the survival. Light, referred also as photic zeitgeber (zeitgeber from German *“time givers”*), is the main external stimulus that regulates autonomous circadian oscillations (4), but food (5), exercise (6), and social activities (7) also act as zeitgeber (**Figure 1** ). In humans, sleep–wake cycle, the best known circadian rhythm, is characterized by a long diurnal active period and a shorter nocturnal sleeping time (8).

Individual differences in sleep–wake rhythm identify different chronotypes (9): morning chronotype (morningness) describes the preference to wake up early and to achieve physical and intellectual peak during the morning, while evening chronotype (eveningness) is typical of subjects that wake up late and prefer work in the later part of the day. Most individuals are characterized by an intermediate chronotype (10–12).

Emerging evidence has indicated that genetic component, gender, and age affect individual chronotype (13–15). In particular, numerous data obtained using animal models and performed clinical studies have demonstrated how, before menopause, women are more frequently associated with morning chronotypes (16, 17). Menopause is related to circadian abnormalities and sleep disturbance (18, 19).

Moreover, in developed countries, socioeconomic organization contributes to the modification of circadian rhythm. In details, the extension of working hours to night with shift work, the different time of food consumption, the artificial light, and, in the last two decades, the use of electronic media (internet/mobile phones/electronic gaming/on demand television) enhance the disruption of circadian cycle (20–22). A large number of individuals, adolescents and above post-adolescent individuals, usually prefer nocturnal activities during weekends, while during weekdays, they follow a different sleep–wake cycle (23–25). The result of this discrepancy between biological and social time, work, and free days is sleep deprivation. This condition, defined as “social jetlag” by Till Roenneberg, is an important cause involved in the onset of obesity and metabolic and cardiovascular diseases (26).

Emerging data highlight a bidirectional relationship between circadian misalignment and cardiometabolic health: circadian disruption promotes the onset of obesity and its comorbidities, including diabetes and cardiovascular diseases; on the other hand, these pathologies exacerbate circadian alterations creating a vicious cycle (27–29). The close interconnection between circadian system and cardiometabolic state assumes a communication network, capable of sending mutual feedbacks from circadian system to different organs and *vice versa*, exists (30–32). This communication network integrates peripheral signals, such as glucose levels, lipid absorption, or blood pressure oscillations, guaranteeing metabolic homeostasis (27).

In this review, we briefly analyzed the principal characteristics of the circadian system focusing our attention on biomolecules, in particular hormones, adipokines, and hepatokines, that join in the communication network and act as synchronizing signals that regulate circadian rhythm and eating. Moreover, we also discussed innovative nutritional strategies aiming at the re-synchronizing of the circadian clock and proposed as a new therapeutic approach to attenuate cardiometabolic pathologies.

## The circadian system: Molecular signaling

The circadian system, responsible for rhythmicity and synchronization of physiological functions, is organized into two different compartments: the central clock, also called central circadian pacemaker or master clock, and several peripheral clocks that regulate daily physiological fluctuations of different tissues (32, 33).

Neurons and glia localized in the suprachiasmatic nucleus (SCN), a tiny region of the hypothalamus above the optic chiasm, play the role of the master clock (34). As previously reported, light represents the fundamental activator of SCN. Classically, melanopsin, a member of the G-protein-coupled receptor family, is a fundamental player involved in SCN activation. Indeed, melanopsin, expressed by photosensitive retinal ganglion cells, acts a photopigment and converts photic energy into a neural signal (4, 35). Recently though, different sophisticated studies have demonstrated that classic retinal photoreceptors, namely, rod and cone photoreceptors, are also implicated in transmission of photic information to SCN (36, 37). Accumulating data suggest that rod photoreceptors are responsible for the SCN stimulation under low light conditions, while input from cones to SCN is essential during twilight transitions, characterized by a shift to shorter wavelengths (38). Additionally, UV-sensitive cone photoreceptors induce SCN neuron response, without melanopsin and rod photoreceptor signaling (37). Therefore, the conversion of photic energy into a neural signal is a combined result of different inputs from all three photoreceptor classes, namely, melanopsin, rod, and cone (4) (**Figure 1A**).

A complex neural network, existing mainly between SCN and the other hypothalamic nuclei in addition to areas of the thalamus, midbrain, and hindbrain, facilities the integration of retina stimuli with other inputs, such as feeding, body temperature, and blood pressure alterations (8, 39, 40).

In human, SCN action is partially mediated by neurohormone melatonin, mainly secreted during darkness by pineal gland and for reason usually called night hormone. If the absence of light promotes melatonin production, melanopsin inhibits it (41). Light wavelength is a crucial parament for the inhibition action of melanopsin; indeed, artificial light inhibits it, while short light wavelength, i.e., blue light, promotes it (42–44). This aspect is extremely relevant considering that in our modern society, blue light is becoming progressively more prominent (45, 46).

Tissues rhythmicity is controlled not only by the SCN but also by specific peripheral clocks capable of combining SCN inputs with several different signals, including food intake, temperature, sympathetic and parasympathetic innervations, and endocrinological and inflammatory signaling (**Figure 1A**).

In both compartments, master and peripheral clocks, a complex transcription–translation feedback loops (TTFLs) generate the circadian rhythm (**Figure 1B**).

The core of this molecular machine is formed by two transcription factors present in all cells of the body and respectively called circadian locomotor output cycles kaput (CLOCK) and brain and muscle ARNT-like 1 (BMAL1) that bind to E-boxes in the promoter region of genes. Period circadian regulators 1–3 (*PER1*, ID:5187; *PER2*, ID:8864; and *PER3*, ID:8863) and cryptochrome circadian regulators 1 and 2 (*CYR1*, ID:1407; *CYR2*, ID:1408) enhance their transcription (47–50) (**Figure 1B**).

PER-CYR protein heterodimers translocate into the nucleus and repress CLOCK-BMAL1 activity. When PER and CYR levels are sufficiently low, heterodimers are degraded, and a new cycle restarts. In normal condition, during day/wake time, CLOCK-BMAL1 heterodimer is active, while in night/sleep time, CLOCK-BMAL1 complex is repressed (51). Different studies have pointed out how the cellular redox state, i.e., the ratio between the oxidized and reduced form of adenine dinucleotide (NAD^+^/NADH), regulating the deacetylase activity of sirtuins, plays a critical role in TTFLs system. SIRT1 and SIRT7 directly deacetylate BMAL1 and CRY, and their activity fluctuates daily (52–55). Moreover, CLOCK-BMAL1 also induces the expression of the nuclear receptors reverse erythroblastoma-erb*α*/β (*REV-ERBα*, ID:9572; *REV-ERBβ*, ID:9975) and retinoic-acid-related orphan genes (*RORα*, ID:6095, *RORβ*, ID:6096; and *R*ORγ, ID:6097) that respectively act as inhibitor and activator of *BMAL1* (ID:406) expression (56, 57). This complex molecular network controls the expression of several genes (**Figure 1B**).

## The circadian system: An orchestra of signals synchronizes feeding and cardiac function

Recent data indicate that in mammalians, 3%–16% of all mRNA display circadian expression and, in particular, 6%–10% hepatic mRNA and 10%–20% of white adipose mRNA have rhythmic daily expression (58–61). This circadian expression primarily affects hormones, enzymes, and transcription factors involved in macronutrient intake and metabolism, as comprehensively discussed by Brubaker et al. in a recent review (62).

Cyclic food accessibility acts as zeitgeber as light/dark cycle, and already at the beginning of the last century, Richter observed increased locomotion in rats during the hours preceding food intake (63). This phenomenon, called food anticipatory activity (FAA), is an important component of circadian rhythms but is not directly regulated by SCN activity, as demonstrated by studies performed using animal models with SCN ablation (64–66). FFA is generated by food-entrainable oscillators (FEOs) whose brain localization is unknown (67).

Moreover, food intake is mainly controlled by carbohydrates, fat, and protein, and by hedonic factors, from taste and appearance to social and emotional factors. Homeostatic stimuli generated by peripheral tissues are integrated by the arcuate nucleus (ARC), the periventricular nucleus (PVN), and the lateral hypothalamus, while mesolimbic circuits, formed mainly by the dopaminergic neurons of the ventral tegmental area (VTA) and the nucleus accumbens (NAc), receive hedonic inputs (39, 68–70). Thus, circadian food intake is a complex result of central signals produced not only by SCN neurons but also by FEOs and by peripheral energetic inputs.

Not only food intake but also numerous cardiac parameters have a peculiar daily rhythm: blood pressure and heart rate are characterized by daily fluctuations (71–73). Moreover, numerous epidemiological studies have revealed that various adverse cardiac events, i.e., myocardial ischemia, sudden cardiac death, and ventricular fibrillation, occur more frequently during the morning (74–76). At molecular levels, different components of the cardiovascular system express circadian genes from smooth muscle cells (77, 78) to cardiomyocytes (79, 80). In addition, different studies performed using murine models have indicated that about 13% of cardiac genes and 8% of cardiac proteins have a circadian expression pattern (81, 82). It is important to note that several data, obtained using mice having mutant circadian genes only in the heart, have demonstrated that heart rate, cardiac metabolism and contractility, and cardiac function are mainly synchronized by the cardiomyocyte circadian clock and not by SCN (83–86). But as observed by Scheer et al., natural light exposure raises sleeping heart rate, emphasizing the role of master clock on cardiac homeostasis (87). Moreover, Curtis et al. have demonstrated that in mice, *BMAL1* gene knockout abolishes the circadian rhythm of blood pressure and contemporarily reduces production of catecholamines (88). Recently, Sedova et al. have tested melatonin integration as therapeutic treatment against ischemia/reperfusion injury (89).

In the next sub-sections, we will examine the action of main biomolecules involved in the circadian regulation of feeding and cardiometabolic processes (**Figure 1**).

### Orchestra of signals: Leptin

Leptin, the most well-known adipokine, is mainly synthesized by white adipocytes and rhythmically secreted during the day with a peak during sleep/inactive phase (90, 91). Leptin’s circadian rhythm is controlled both by SCN, through sympathetic inputs to adipocytes (92); moreover, recently, Luo et al. have demonstrated how dopaminergic neurons localized at the peri-SCN area contribute to leptin expression (93). In addition, food intake regulates leptin secretion: while meal increases levels of this adipokine, fasting has an opposite effect. The correlation between nutritional status and leptin’s secretion reveals the main function of this hormone: suppressing hunger, as suggested by the Greek origin of its name “leptos,” namely, thin (94, 95). ARC, a major target of leptin, is a complex hypothalamic nucleus having a crucial role on different physiological processes, from feeding to reproduction. ARC functions are guaranteed by specialized neuron subtypes; in particular, control of feeding is mainly ensured by two different neuronal subpopulations having opposite role: proopiomelanocortin/cocaine-amphetamine-related transcript (POMC/CART)-positive neurons transmit anorexigenic inputs, while neuropeptide Y (NPY)-/agounti-related peptide (AgRP)-positive neurons regulate orexigenic stimulus. Leptin binding to its receptor inhibits NPY/AgRP neurons, whereas enhances POMC/CART activity triggering the secretion of anorexigenic peptide alpha-it melanocyte stimulating hormone (α-MSH) (96). Binding between melanocortin 4 receptor (MC4R), expressed on distinct second-order neurons in PVN, and α-MSH suppresses food intake (97). Moreover, leptin also influences hedonic response to food: Domingos et al. have proven that leptin inhibits mesolimbic dopamine neuronal activity, decreasing the reward value of sucrose (98), and recently, Omran et al. have proposed that leptin reduces the pleasure of food activating *via* inhibitory GABA neurons in VTA that directly regulate dopamine neurons (99). It is important to note that satiety induced by the melanocortin system is antagonized by NPY/AgRP neurons. As demonstrated by Ollmann et al., AgRP plays as a competitive antagonist of the MC4R (100).

Furthermore, various studies have highlighted the correlation between plasma leptin levels and hypertension development (101, 102). Interestingly, Han et al. have demonstrated that in normal mice, central administration of a leptin receptor antagonist abrogates diurnal rise of blood pressure, while in obese mice, leptin mediates the diurnal blood pressure elevation, promoting tumor necrosis factor-α (TNFα) signaling (103).

### Orchestra of signals: Ghrelin

Not only leptin but also ghrelin, known as hunger hormone, affects ARC neurons regulating food intake and body composition (104). Ghrelin is primarily secreted by oxyntic cells of the gastric mucosa, and its action is due to acylation process that allows its binding to growth hormone secretagogue receptor (GHSR), highly expressed in ARC (104–106). However, it is important to note that GHSR is differently expressed in ARC neuronal populations: about 90% of NPY/AgRP expressed GHSR compared to ~10% POMC neurons (107, 108). Consequently, ghrelin mainly activates NPY/AgRP neurons promoting food intake (109). Plasma ghrelin levels continuously fluctuate during the day, and nutritional status is the main factor responsible of this fluctuation: fasting raises the activity of oxyntic cells, while food intake plays in opposite manner, decreasing ghrelin levels (105, 110, 111). In addition, in rodents and human, ghrelin’s peak is observed during the inactive phase, while in the active phase of circadian cycle, ghrelin is low (104, 112, 113).

### Orchestra of signals: GIP/GLP-1

In addition, different intestine biomolecules secreted after food intake influence circadian rhythm. In 1932, La Barre hypothesized the existence of these molecules and coined the word “incretin,” and about 40 years later, Dupre et al. discovered the duodenal glucose-dependent insulinotropic polypeptide, GIP, and only in 1987, Holst identified glucagon-like peptide-1 (GLP-1) (114–117). Both GIP and GLP-1 contribute to maintain diurnal euglycemia; GIP promotes insulin production, while GLP-1 also strongly reduces glucagon secretion (118). The circadian release of GLP-1 by enteroendocrine L cells is well established, and GLP-1 peak is observed in the morning at the onset of the active period (dark for mice and light for humans), while circadian GIP secretion by proximal K cells is partially controversial (119–121). Recently, the study performed by Martchenko et al. have demonstrated that in normal male and female mice, both GLP-1 and GIP release are characterized by 24-h rhythm, whereas in obese mice, circadian secretion of GLP-1 is fundamental to control insulin action (122, 123). However, the most important result of these works concerns the role played by intestinal microbiome: performing experiments using antibiotic-induced microbial depletion in germ-free mice, Martchenko et al. observed that diurnal GLP-1 release was regulated by intestinal microbiota (122). This observation is recently confirmed by the study coordinated by Grasset, which showed how GLP-1 release in mice is the result of a specific pattern of clock gene expression, in particular *BMAL1*, and oscillation of some ileum bacteria, including *Lachnospiraceae* (124). Additionally, Grasset et al. have proven the role of master clock on GLP-1 signal: the disruption of gut–brain axis by monolateral subdiaphragmatic vagotomy impairs diurnal rhythmicity of GLP-1 and consequently its action. It is important to note that GLP-1 crosses the blood–brain and, above all, that GLP-1 is synthetized by preproglucagon-neurons (PPG) in the nucleus of the solitary tract (125, 126). PPG neurons project to several nuclei that express the GLP-1 receptor, including hindbrain, hypothalamus, and mesolimbic brain areas. These nuclei are involved in reward circuit (127, 128), and thus, GLP-1 influences hedonic eating, reducing palatable food intake as reported in a recent review presented by Eren-Yazicioglu et al. (129). Furthermore, several emerging data, obtained from treated different animal models with GLP-1 agonists, suggest that GLP-1 signaling plays an important action in drug addiction, i.e., cocaine (130), amphetamine and alcohol (129). Data obtained from studying cocaine abuse are extremely remarkable (130), suggesting that cocaine activates PPG in the nucleus of the solitary tract by an increase in plasma and central corticosterone levels (131). Consequently, the GLP-1 receptor pathway in midbrain and forebrain areas is activated and acts as a negative-feedback response (132). Obviously, further studies focusing on the relationship between GLP-1 central signaling–corticosterone axis and seeking behaviors must be performed. In any case, GLP-1 signaling plays an important role in psychological stress responses.

### Orchestra of signals: FGF-21

In addition to adipokines and gastrokines, hepatokines are also mediators of the circadian rhythm. Different nutritional inputs affect the circadian secretion of fibroblast growth factor 21 (FGF-21), a hepatokine discovered in 2000, which is secreted by other tissues from adipose tissue to skeletal muscle (133, 134). Several researchers, including Yu et al., have observed that circulating FGF-21 levels have a characteristic diurnal rhythm in humans peaking around midnight (135, 136). As other biomolecules, food intake influences FGF-21 secretion; in particular, prolonged hunger, protein restriction, or carbohydrate-rich diets potently stimulate circulating FGF-21 in humans (137). Transcription factor peroxisome proliferator-activated receptor α (PPARα) is the main mediator of different nutritional states; in fact, PPARα binding *FGF21* (ID: 26291) promotor region enhances its expression (138–140). Metabolically circulating FGF-21 stimulates insulin-dependent glucose uptake in peripheral tissues, preventing excessive hyperglycemia after food intake (141–143). Interestingly, several studies also performed using transgenic mice models have shown how FGF-21 decreased sweet and alcohol intake, acting on hypothalamic neurons and regulating food intake. Matsui et al. have revealed that FGF-21 acts as a sweet-intake inhibitor on hypothalamic oxytocin neurons (144), while Jensen-Cody et al. have demonstrated that FGF-21, after binding to its receptor, β-Klotho (KLB) co-receptor, activates glutamatergic neurons in the ventromedial hypothalamus (VMH), suppressing sweet intake (145). However, KLB receptor is also expressed in the SCN and in the dorsal vagal complex of the hindbrain, and Bookout et al., using different mouse models, have shown that the binding of FGF-21 to its receptor in the brain enhances an adaptive starvation response, characterized by an increased systemic corticosterone level, an alteration of metabolism and light/dark cycle activity (146). Additionally, Bookout et al. have observed that in the SCN, the expression of the neuropeptide vasopressin is suppressed by FGF-21 (146).

### Orchestra of signals: AVP and cortisol

Vasopressin (AVP) is a neurotransmitter secreted by ~20% of SNC neurons, and AVP neurons are important components of circadian pacemaker (147–149). CLOCK/BMAL1 complex binds the *AVP (ID:551)* promoter region and induces AVP synthesis (150, 151), while CRY1 and CRY2 repress it (152). AVP function strictly correlates with metabolic and behavioral rhythm of the first hours of active and inactive phases. In fact, activation of SCN-AVP neurons, projecting to the organum vasculosum lamina terminalis, influences the anticipatory thirst prior to sleep (153). But above all, AVP promotes cortisol peak before awakening.

In humans, cortisol is secreted by adrenal glands in a circadian manner even if different stress conditions modify its secretion (154). In normal conditions, cortisol levels rise at the end of the sleep phase, reaching the peak in the first hour after waking; this crucial point is known as “cortisol awakening response,” underlining the role of cortisol in the beginning of the active phase (154, 155). Cortisol expression is directly regulated by adrenocorticotrophic hormone (ACTH) whose secretion is controlled by AVP and corticotropin-releasing hormone (CRH), produced by the hypothalamic paraventricular nucleus (156, 157). It is important to note that in both nocturnal and diurnal animals, AVP is released during the light period, but AVP action is different: in nocturnal animals, like mice, AVP inhibits CRH neurons, while in diurnal animals, AVP promotes them (158). Moreover, by a negative feedback mechanism, high cortisol levels inhibit CRH, AVP, and ACTH expression, whereas low cortisol levels act in opposite manner. This mechanism is altered by physical and psychological stress factors. The physiological role of cortisol is extremely well-structured, which involves not only energetic metabolism (breakdown of carbohydrates and inhibition of gluconeogenesis) but also immune processes and, above all, cardiac function (159–161).

### Orchestra of signals: GDF15

Interestingly, in recent years, many researchers have correlated cardiac dysfunction to the circulating level of growth differentiation factor 15 (GDF15), a peptide hormone member of the transforming growth factor β superfamily, which is expressed in several tissues, from kidney to adipose tissue (162). Tsai et al. have observed that in normal state, GDF15 is expressed at a low concentration with a diurnal variation that is not directly related to meals (163), but as demonstrated by Zhao et al., circadian expression of GDF15 is correlated with inhibition of REV-ERBs (164). In pathological conditions, mainly characterized by inflammation state and mitochondrial dysfunction, GDF15 expression is overexpressed. High levels of GDF15 have been detected in obese subjects and in patients inflicted with different cardiovascular diseases (162, 165, 166). Accumulating data suggest a crucial role of GDF15 on the control of food intake. The first evidence of the possible correlation between weight and GDF15 has been obtained by studying individuals with advanced prostate cancer: in these subjects, high circulating GDF15 levels correlated with weight loss (167). In rats fed a high-fat diet, GDF15 treatment suppresses food intake, enhances body weight loss, and consequently ameliorates cardiometabolic condition (168). Accumulating data indicate that GDF15 modifies appetite through multiple systemic mechanisms, including changes in food preferences, gastric emptying, and nausea, but at cellular level, GDF15 action is exclusively mediated by GDNF family receptor α-like (GFRAL) (162, 169–173). Indeed, in GFRAL knockout mice, anorectic effects of GDF15 are inhibited; GFRAL^−/−^ mice are hyperphagic under stressed conditions, refractory to the effects of recombinant human GDF15 on body weight, food intake, and glucose parameters and are resistant to chemotherapy-induced anorexia (171, 174). It is important to note that GFRAL is expressed in hindbrain neurons, specifically in the nucleus of the solitary tract and the area postrema (173–175). As demonstrated by Hsu et al., the parabrachial nucleus and the central amygdala, which are circuits that regulate food intake and body weight under stressed conditions, are the primary neural circuits that respond to GFRAL activation (176). Thus, it is possible to speculate that GDF15 is a stress biomarker. In line with this hypothesis, epidemiological data obtained by studying patients affected by chronic cardiac pathologies, i.e., hypertrophy and endothelial dysfunction, have demonstrated that a high level of GFD15 is a crucial biomarker associated with unfavorable prognosis (165, 166). In mouse models of ischaemia–reperfusion injury, GDF15 deficiency correlated with a major damage and cardiomyocyte apoptosis, while treatment with GDF15 counteracts ischaemia–reperfusion injury (177–179). This positive action of GDF15 treatment in obese and in ischemia-reperfused mice suggest that high levels of GDF15 play a compensatory role as recently suggested by Townsend et al., who demonstrated that the increase in hepatic GDF15 is associated with the energy stress response controlled by AMPK (180). Additionally, Patel et al. demonstrated that different nutritional stress conditions, such as prolonged high-fat diet or an amino acid imbalanced diet, induce a significant increase in circulating GDF15 levels (174). Recently, Miyake et al. observed that in adipocytes, GDF15 action is correlated with the eIF2α phosphorylation-dependent integrated stress response (ISR), a signaling pathway involved in the maintenance of cellular homeostasis exposed to different stresses (181). Finally, several data have shown that GDF15 are involved in mitochondrial and endoplasmic reticulum stress conditions in non-alcoholic steatohepatitis (182).

## When the orchestra is out of tune: Circadian misalignment/desynchronization

Like an orchestra, this complex system of communication can be out of tune: circadian misalignment is an important risk factor for the development of cardiometabolic pathologies (**Figure 1**).

As mentioned in *Introduction*, different causes induce a disharmony between central clock and peripheral clocks. Shift work, jetlag, and social jetlag are probably the most studied external factors able to disturb circadian rhythmicity (1, 7, 20, 22, 24, 26).

In addition, eating contributes to circadian disturbances (5). Yasumoto et al. have fed mice during their inactive phase, i.e., daytime for nocturnal animals like mice (daytime feeding with high-fat/high-sucrose diet, DF), and have observed desynchronization of peripheral clocks and, above all, obesity onset. Moreover, mice were affected by hepatic fat accumulation and impaired leptin signaling (183). Leptin signaling appears crucial in circadian misalignment induced by DF: Oishi et al. have shown that DF-induced metabolic damages were abolished in mice expressing mutations in leptin receptor (db/db mice) (184). In addition, not only the adipose tissue and liver are targets of DF, but the skeletal muscle is also injured: Abe et al. revealed that DF reduced skeletal muscle mass in mice (185).

Inappropriate eating behaviors disrupt cardiometabolic homeostasis also in humans. As previously reported, shifts workers are more predisposed to obesity, diabetes, and cardiovascular diseases than day workers (186). Additionally, data obtained by several population-cohort study have proven how a higher energy intake during evening/night increases the risk of metabolic syndrome and cardiovascular disease (187–191). During the coronavirus disease 2019 (COVID-19) pandemic, young people have accentuated this tendency to consume sweet foods/snacks in the evening hours. Furthermore, adolescents were exposed to lengthier screen time and have shown an inadequate sleeping pattern (192, 193). Woo et al. have identified that these changes in lifestyle behaviors are associated with weight gain and with worsening of some cardiometabolic markers, including triglycerides and leptin, in a cohort of children and adolescents with overweight and obesity (194).

In addition, subjects affected by night eating syndrome (evening hyperphagia/nocturnal ingestions) usually are characterized by circadian phase delays of leptin, cortisol, and melatonin. These abnormalities are associated with a higher risk of obesity and consequently of cardiovascular disease (195, 196). It is important to note that these patients prefer eating fast food and sugar-sweetened beverages (196).

In fact, not only the time of eating but also the composition of meal is involved in cardiometabolic damages (5, 197–199). In the past decades, studies performed using animal models have revealed that a high-fat diet alters the expression and cycling of circadian clock genes and clock-controlled genes involved in metabolic metabolism in different murine tissues, including the hypothalamus, liver, and adipose tissue (200–202).

In addition, the National Health and Nutrition Examination Survey 2003–2016 (NHANES) study, which included a total of 27,911 participants, showed that an excessive intake of low-quality carbohydrates and animal protein at dinner was significantly connected with higher cardiovascular disease risk (203, 204). Accumulating data have corroborated these observations in humans (205), and recently, Kessler et al. have demonstrated that a diet characterized by fat-rich meals until 13:30 and carbohydrate-rich meals between 16:30 and 22:00 worsens metabolic condition in subjects with impaired glucose metabolism (206).

Finally, it is important to note that dietary cues are bidirectionally correlated with gut microbiota, including bacteria, yeasts, and viruses. Firmicutes and Bacteroidetes species represent 90% of the gut microbiota even if Actinobacteria, Proteobacteria, Fusobacteria, and Verrucomicrobia bacteria are presented. As demonstrated by a large amount of data, unbalanced intestinal ecosystem, in particular the ratio between Firmicutes and Bacteroidetes, is related to obesity (207). Microbial components, primarily lipopolysaccharide, and specific microbial metabolites, short-chain fatty acids (SCFAs), and unconjugated bile acids, influence the expression of gene clocks in peripheral tissues and, above all, in intestinal epithelial cell (208, 209). Tahara et al. have demonstrated that oral administration of mixed SCFAs and lactate positively influences PER2 in peripheral tissues (210). Mukherji et al., after disrupting murine microbiota by antibiotics, have observed an overproduction of corticosterone, synthetized by intestinal epithelial cells, and an impaired expression of circadian genes. But above all, Mukherji et al. have determined that after a month, mice developed prediabetic syndrome (211). At the same time, feeding/starvation rhythm and food composition alter gut composition and consequently induced desynchronization (212). Mice fed a normal diet are characterized by cyclical fluctuation in gut microbial composition: Firmicutes species reach their abundant peak during dark/active phase, whereas Bacteroidetes and Verrucomicrobia species have an opposite cycle with peak during light/rest phase (213, 214). Conversely, mice fed a high-fat diet destroys microbial diurnal cyclical fluctuation, causing a drastic reduction in the diversity and variability of microbial communities. A high-fat diet modifies gut microbiota by enhancing Firmicutes species, which express more enzymes involved in carbohydrates and lipids metabolism (212, 213). In addition, a high-fat diet is associated with the production of microbially derived metabolites, especially short-chain fatty acids, that are able to increase the expression of *BMAL1* gene in the hypothalamus, as also demonstrated by Leone et al. (215).

## Nutrition as metronome: Circadian re-synchronization

However, meal timing and composition is not only a problem for cardiometabolic homeostasis but also a fundamental solution. In fact, nutrition could be a crucial metronome that is able to lead circadian re-synchronization (**Figure 1**). Observational data have revealed that meal regularity and, in particular, high energy intake in the morning is associated with wellbeing outcomes (216). It is important to note that food timing also influences the metabolic state of obese subjects: as observed by Mazri et al., early temporal patterns of energy and macronutrient intake characterized obesity with healthy metabolic status (217). In diabetic patients, morning distribution of food intake, especially a carbohydrate-rich breakfast, accelerates weight loss, reduces appetite and craving, and thus improves metabolic parameters, such as postprandial glycemia and glycated hemoglobin (218–220). Moreover, Jakubowicz et al., after a nutrition study performed in 193 obese healthy patients, suggested that a high energy breakfast, primarily composed by carbohydrates and proteins, could prevent obesity relapse by decreasing hunger/craving and influencing ghrelin signaling. Molecular studies have highlighted that the absence of breakfast is associated with an inhibition of clock genes expression, in particular *BMAL1* and *PER* genes, whereas a high-energy breakfast has an opposite effect (221, 222).

In the last decades, several researchers have studied in animal models the effects of time-restricted feeding (TRF), called time-restricted energy (TRE) if referring to humans, as therapeutic treatment aimed to re-synchronize and consequently prevent cardiometabolic damage. This daily nutritional intervention is characterized by the restriction of food consumption to certain hours, while the daily fasting period lasts >12 h without modifying nutrient quality or quantity (223, 224).

Using different animal models, several studies were performed to investigate TRF action. Usually, these experiments were designed to compare the metabolic state of mice having access to food (chow or high fat) *ad libitum* with mice having access to food restricted to 8–10 h. Chaix et al. studied TRF action on a particular knockout mouse, lacking whole-body *CRY* gene and *BMAL1* and *REV-ERBα/β* in the liver. In both animal models, TRF diet protected mice from excessive weight gain and metabolic diseases. Moreover, TRF significantly reduced hepatic lipid accumulation and improved antioxidant cellular defenses. It is important to underline that these results indicate that TRF action is independent by clock gene expression (225). Accumulating data have revealed that also in mice fed a high-fat diet, TRF has shown beneficial effects counteracting excessive weight gain and protecting from metabolic diseases. TFR has improved nutrient utilization and energy expenditure, restoring expression oscillations of the circadian clock genes and daily rhythms of ghrelin and corticosterone (226, 227). In addition, TRF has improved murine gut microbiota, re-establishing cyclical variation in many families of bacteria including *Lactobacillus* family (228). In addition, in *Drosophila melanogaster*, this dietary approach ameliorates cardiometabolic state attenuating age-related cardiac deterioration (229) and enhances muscle performance by decreasing intramuscular fat deposits in obese model of this insect (230).

Interestingly, results obtained by human trials are encouraging (231). Meta-analysis performed by Moon et al. have shown how TRE positively influences body weight in overweight or obese patients, reducing fat mass and preserving fat-free mass, and ameliorates metabolic parameters, including blood pressure, fasting glucose concentration, and cholesterol profiles (232). Clinical trials performed by Sutton et al. corroborates TRE-induced cardiometabolic improvement even if, in this cohort of men with prediabetes, TRE did not modify weight (233). Similarly, Jones et al. obtained comparable results in healthy men treated with TRE for 2 weeks: they enhanced whole-body insulin sensitivity and skeletal muscle glucose and branched-chain amino acids uptake without modifying body weight (234).

Taken together, data obtained by human studies point out that TRE has several beneficial impacts on the cardiometabolic state in healthy and obese patients (235). Moreover, in obese patients TRE seems to promote body composition remodeling, emphasizing food timing–circadian cycle interconnection. It is important to note that results obtained by human trials have shown that TRE is safe and well-tolerated with good adherence (235).

Additionally, nutraceutical compounds or functional foods could represent an effective strategy to normalize circadian rhythm. The action of polyphenols, in particular resveratrol, in cardiometabolic pathologies are well investigated by *in vitro* and *in vivo* models (236–238). Moreover, resveratrol and other polyphenols are able to modify gut composition, ameliorating metabolic abnormalities, including liver steatosis and insulin resistance (239, 240). But above all, resveratrol is able to activate SIRT1, an important mediator of circadian molecular pathway, as previous described (241, 242). Based on these data, researchers have tested the possible action of resveratrol, and in general polyphenols, on re-synchronization (243). As previously reported, these studies have been performed *in vitro* or using different animal models; not rarely, resveratrol is tested in association with other nutraceuticals. For these reasons, many aspects correlated to therapeutic use of resveratrol as chronobiotic agent are not completed clarified, i.e., molecular mechanism on circadian genes or the effective dose. In any case, the preliminary data are more promising. Li et al. observed that resveratrol mitigates impaired intracellular lipid metabolism in a BMAL1-dependent manner in hepatocytes (244). In addition, Sun et al. investigated the effects of resveratrol supplementation on high-fat-diet-induced disorders in mice, observing a significative decrease in body weight and a rhythmic restoration of fasting blood glucose and leptin associated with different expression pattern of *CLOCK*, *BMAL1*, and *PER2* genes (245). Similar results have been obtained by Koh et al., who treated jetlagged mice with pterostilbene and resveratrol. In these animals, the combination of pterostilbene and resveratrol improved gut diversity (246).

Considering the relationship between gut and circadian rhythms, recently, several authors have begun to examine prebiotic supplementation as dietary approach for mitigating circadian misalignment. In obese mice exposed to weekly shifted light–dark cycle, β-glucan and inulin supplementation have restored the expression and phase of circadian-clock genes (247). Comparable effects have been achieved using oat β-glucan, which is able to improve microbial gut diversity and to counteract body weight gain and leptin signaling alteration restoring glucose tolerance (248). Obviously, there are other nutraceutical compounds that could influence circadian machine regulating intestinal flora and bioactive molecules secretion (249). Recently, Huang et al. proposed several nutraceutical molecules as chronobiotics (250): first data are promising but, as previously reported, were mainly obtained *in vitro* and in animal models, and therefore, it will be necessary to perform robust human studies especially in order to evaluate the bioavailability of these compounds, for example, resveratrol and other polyphenols characterized by low bioavailability. Moreover, it will be important to design closer investigations for specific diseases, such as different cardiovascular pathologies, to better understand extensive therapeutic action of different nutraceutical supplements.

## Conclusion

Growing evidence demonstrates that circadian rhythm is a crucial aspect to ensure cardiometabolic health. Circadian rhythm is a complex network able to interconnect different signaling: from photic zeitgeber to food, exercise, and social behaviors. Bioactive compounds secreted by tissues are mainly responsible for daily inter-organ communication. However, as an orchestra, this system can be out of tune leading to obesity and metabolic and cardiovascular diseases development. Eating, mainly altered meal timing and composition, causes circadian dissonance. Recently, however, interesting studies seem to suggest that eating could play as a chrono-regulator promoting re-synchronizing circadian rhythm. Data obtained mainly using *in vitro* and animal models indicate that high-calorie breakfast associated with reduced food intake at dinner or supplementation with nutritional compounds could regulate the expression of circadian clock transcription factor or the composition of gut microbiota. But above all, TRE could counteract circadian misalignment beneficially influencing cardiometabolic state. The results obtained from the first human trials are encouraging and support the hypothesis that specific nutritional treatments could positively influence circadian misalignment: high-energy breakfast and TRE have beneficial effects and, above all, are well accepted by patients.

Nevertheless, considering the short duration of the clinical trials and/or the small sample size of enrolled subjects, further studies will be indispensable to well establish the clinical efficacy of different nutritional interventions and to standardize the treatment protocols. To this regard, clinical trials based on the use of mobile app should be encouraged considering the rapid development of e-health in nutrition clinical practice.

Even if many aspects of the relationship between circadian rhythm, secreted biomolecules, and nutrition have yet to be elucidated, the translation of ancient maxim, “Eat breakfast like a king, lunch like a prince and dinner like a pauper,” in daily nutritional therapy could represent an additional medical intervention in the management of cardiometabolic pathologies.

## Author contributions

PS and IT designed the article. PS wrote the manuscript. AF and LL revised the manuscript. All authors contributed to the article and approved the submitted and the published version.

## Funding

This work has been supported by Ministry of Health—Ricerca Corrente—IRCCS MultiMedica.

## Conflict of Interest

The authors declare that the research was conducted in the absence of any commercial or financial relationships that could be construed as a potential conflict of interest.

## Publisher’s note

All claims expressed in this article are solely those of the authors and do not necessarily represent those of their affiliated organizations, or those of the publisher, the editors and the reviewers. Any product that may be evaluated in this article, or claim that may be made by its manufacturer, is not guaranteed or endorsed by the publisher.

## Abbreviations

SCN, suprachiasmatic nucleus; TTFLs, transcription–translation feedback loops; CLOCK, circadian locomotor output cycles kaput; BMAL1, brain and muscle ARNT-like 1; PER1–3, period circadian regulators 1–3; CYR1/2, cryptochrome circadian regulators 1 and 2; REV-ERBs, nuclear receptors reverse erythroblastoma-erbα/β; ROR, retinoic-acid-related orphan; NAD+, oxidized form of adenine dinucleotide; NADH, reduced form of adenine dinucleotide; FAA, food anticipatory activity; FEOs, food-entrainable oscillators; ARC, arcuate nucleus; PVN, periventricular nucleus; VTA, ventral tegmental area; NAc, nucleus accumbens; POMC/CART, POMC-/cocaine-amphetamine-related transcript (CART); NPY/AgRP, neuropeptide Y/agounti-related peptide; α-MSH, alpha-melanocyte stimulating hormone; MC4R, melanocortin 4 receptor; TNFα, tumor necrosis factor-α; GHSR, growth hormone secretagogue receptor; GIP, duodenal glucose-dependent insulinotropic polypeptide; GLP-1, glucagon-like peptide-1; PPG, preproglucagon-neurons; FGF-21, fibroblast growth factor 21; PPARα, peroxisome proliferator-activated receptor α; KLB, β-Klotho co-receptor; VMH, ventromedial hypothalamus; AVP, vasopressin; ACTH, adrenocorticotrophic hormone; CRH, corticotropin-releasing hormone; GDF15, growth differentiation factor 15; GFRAL, GDNF family receptor α-like; SCFAs, short-chain fatty acids; TRF, time-restricted feeding; TRE, time-restricted energy.
